# Elaboration, structural study and validation of a new NASICON-type structure, Na_0.72_(Cr_0.48_,Al_1.52_)(Mo_2.77_,Al_0.23_)O_12_

**DOI:** 10.1107/S2056989018003031

**Published:** 2018-02-28

**Authors:** Manel Sonni, Imen Jendoubi, Mohamed Faouzi Zid

**Affiliations:** a University of Tunis El Manar, Faculty of Sciences of Tunis, Laboratory of Materials, Crystal Chemistry and Applied Thermodynamics, 2092 ElManar II, Tunis, Tunisia

**Keywords:** NASICON structure, framework, crystal structure, BVS, CHARDI

## Abstract

The title compound, sodium chromium/aluminium molybdenum/aluminium dodeca­oxide, Na_0.72_Cr_0.48_Al_1.74_Mo_2.77_O_12_, was prepared by solid-state reaction. Its crystal structure is related to NaSICON-type compounds. The framework is built up from *M*1O_6_ (*M*1 = Cr/Al) octa­hedra and *M*2O_4_ (*M*2 = Mo/Al) tetra­hedra inter­connected by corners. The three-dimensional framework contains cavities in which sodium cations are located. Two validation models (BVS and CHARDI) were used to confirm the proposed structural model. The mobility of Na^+^ ions in the structure has been investigated by theoretical means.

## Abstract 2

A new NASICON-type phase, Na_0.72_(Cr_0.48_,Al_1.52_)(Mo_2.77_,Al_0.23_)O_12_, was synthesized by solid-state reaction. The structural unit consists of one octa­hedron *M*1O_6_ (*M*1 = Cr1/Al2) and one tetra­hedron *M*2O_4_ (*M*2 = Mo1/Al1) sharing corners. The charge compensation is provided by Na^+^ cations.

## Chemical context   

The search for new and better solid electrolyte materials has grown considerably in recent years because of their amazing properties and their diverse applications in the field of solid-state chemistry. Indeed, many new molybdate phases with high ionic conductivity have been synthesized and structurally characterized by X-ray diffraction. A large number belong to the NASICON (‘Na super ionic conductor’) family, *e.g*. phosphate (Tkachev *et al.*, 1984 ▸; Catti *et al.*, 2004 ▸), arsenate (Harrison & Phillips, 2001 ▸), sulfate (Slater & Greaves, 1994 ▸) and molybdate (Sun *et al.*, 2012 ▸; Kozhevnikova & Imekhenova, 2006 ▸) based systems. The NASICON family groups together a set of phases of the same structural type with the general formula *AM*_2_(*X*O_4_)_3_ (*A* = alkali, *M* = Ti, Fe, V, Co, Ni and *X* = P, As, Mo, W, S; Prabaharan *et al.*, 2004 ▸). Apart from their superionic properties, a number of NASICON compounds have considerable potential for use in laser engin­eering, optics and electronics owing to their non-linear optical, electrical, magnetic and luminescent properties. It is in this context that we chose to explore *A*–Cr–Mo–O systems (*A* = monovalent ion). A new phase Na_0.72_(Cr_0.48_,Al_1.52_)(Mo_2.77_,Al_0.23_)O_12_ was synthesized by solid-state reaction. We present here its crystal structure and its validation by the CHARDI (charge distribution) and BVS (bond-valence-sum) methods.

## Structural commentary   

**Figure 1:**
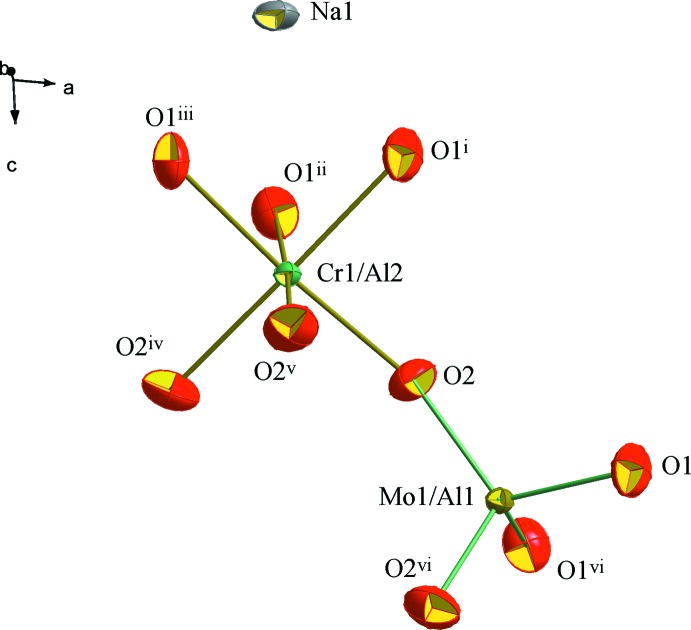
Structural unit of Na_0.72_(Cr_0.48_,Al_1.52_)(Mo_2.77_,Al_0.23_)O_12_. Displacement ellipsoids are drawn at the 50% probability level. [Symmetry codes: (i) 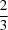 − *x*, 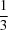 − *y*, 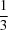 − *z*; (ii) −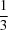 + *x* − *y*, −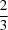 + *x*, 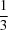 − *z*; (iii) −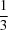 + *y*, 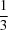 − *x* + *y*, 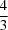 − *z*; (iv) −*x* + *y*, −*x*, *z*; (v) −*x*, *x* − *z*, *z*; (vi) *x* − *y*, −*y*, 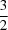 − *z*.]

**Figure 2:**
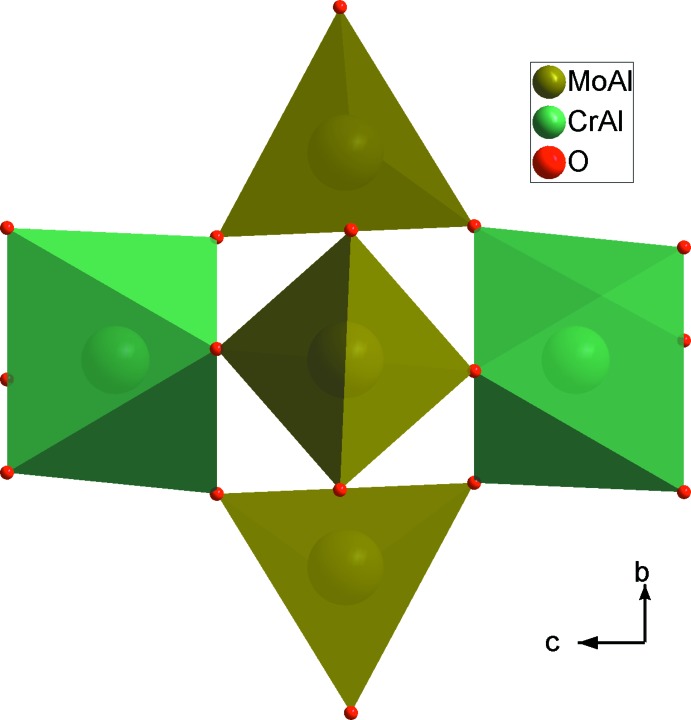
Projection of an *M*1_2_ *M*2_3_O_18_ unit along the *a* axis.

**Figure 3:**
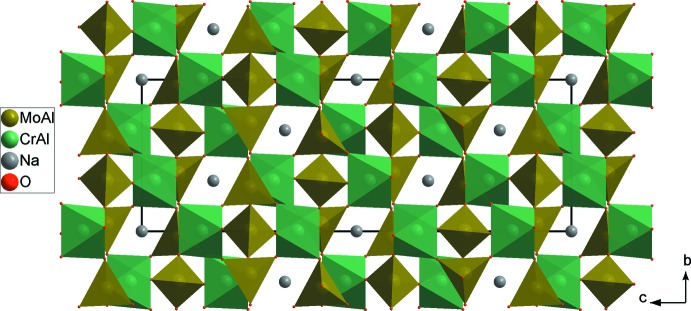
Projection of Na_0.72_(Cr_0.48_,Al_1.52_)(Mo_2.77_,Al_0.23_)O_12_ along the *a* axis.

**Figure 4:**
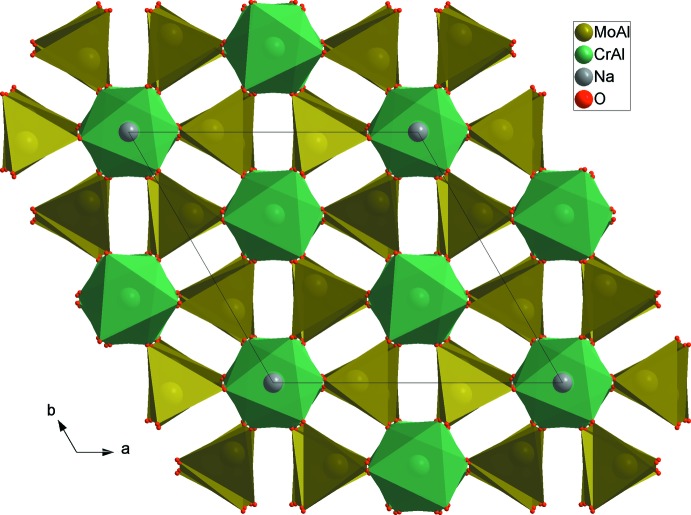
Projection of Na_0.72_(Cr_0.48_,Al_1.52_)(Mo_2.77_,Al_0.23_)O_12_ along the *c* axis.

**Figure 5:**
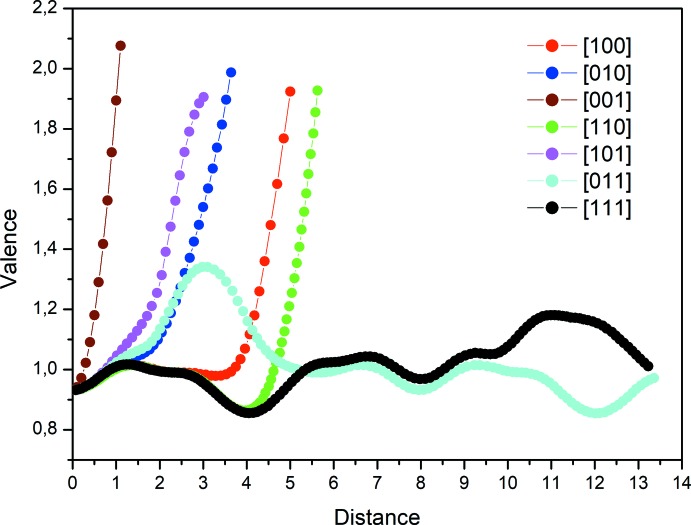
Ionic pathway valence curves of the title compound.

**Figure 6:**
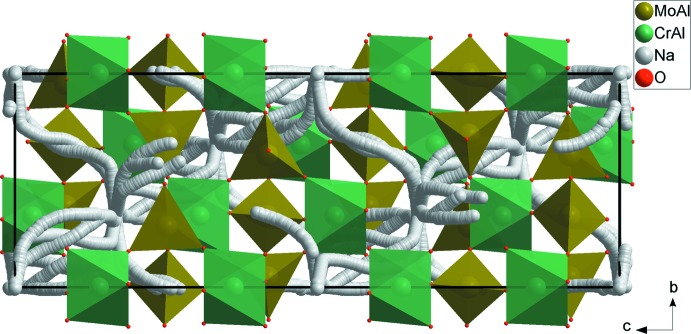
Modelling of the Na^+^ pathway in Na_0.72_(Cr_0.48_,Al_1.52_)(Mo_2.77_, Al_0.23_)O_12_.

The structural unit of Na_0.72_(Cr_0.48_,Al_1.52_)(Mo_2.77_,Al_0.23_)O_12_ consists of one octa­hedron *M*1O_6_ (*M*1 = Cr1/Al2) and one tetra­hedron *M*2O_4_ (*M*2 = Mo1/Al1) that share corners. The charge compensation is provided by Na^+^ cations (Fig. 1 ▸). The main construction unit in the anionic framework of the compound Na_0.72_(Cr_0.48_,Al_1.52_)(Mo_2.77_,Al_0.23_)O_12_ is formed by two *M*1O_6_ octa­hedra inter­connected by three *M*2O_4_ tetra­hedra located along the *c* axis. Geometrical parameters are given in Table 1 ▸. This assembly forms *M*1_2_ *M*2_3_O_18_ units (Fig. 2 ▸). The junction between these units, provided by the formation of mixed bridges of the *M*1–O–*M*2 type, leads to a three-dimensional framework with cavities parallel to the [100] and [010] directions in which the Na^+^ cations are located (Fig. 3 ▸). Indeed, each *M*1O_6_ octa­hedron share its six corners with different *M*2O_4_ tetra­hedra, leading to *M1M2*_6_O_24_ clusters (Fig. 4 ▸). The two validation models BVS (Brown & Altermatt, 1985 ▸; Brown, 2002 ▸; Adams, 2003 ▸) and CHARDI (Hoppe *et al.*, 1989 ▸; Nespolo *et al.*, 2001 ▸; Nespolo, 2001 ▸) confirm the proposed structural model, in particular the distribution at mixed sites. The calculated load values *Q*(*i*) and valences *V*(*i*) are in good agreement with the oxidation degrees weighted by the occupancy rates. The dispersion factor σ_cat_, which measures the deviation of the calculated cationic charges, is equal to 0.3% (Table 2 ▸). The variation of the bond-valence sum of sodium as a function of the distance travelled in different directions shows that the [011] and [111] directions are the most favorable directions for the mobility of sodium. The paths along these directions have the same shape when the distance travelled is about 13.5 Å and the maximum valence is about 1.4 valence units (Fig. 5 ▸). The representation of the Na migration path in the direction [011] is shown in Fig. 6 ▸.

## Database survey   

A comparison between the structures of the title compound and those of other NASICON-type compounds reveals that other compounds also crystallize in the *R* 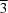 *c* space group with similar unit-cell parameters. When compared to NaZr_2_(AsO_4_)_3_ (Coquerel *et al.*, 1983 ▸) and Na_4_Co_3_Mo_22.33_O_72_ (Chakir *et al.*, 2003 ▸), the only difference observed is the occupancy of the sites 6*b*, 12*c* and 18*e*. In NaZr_2_(AsO_4_)_3_, the sites are fully occupied, whereas in Na_4_Co_3_Mo_22.33_O_72_, the 6*b* site is not totally occupied, and the 12*c* site is occupied by both Co and Mo. In the title compound, the 6*b* site is partially occupied and the 12*c* and 18*e* sites are mixed Cr/Al and Mo/Al sites, respectively.

## Synthesis and crystallization   

During the investigation of the *A*–Mo–Cr–O phase diagrams (*A* = Li, Na, Ag), the new compound Na_0.72_(Cr_0.48_,Al_1.52_)(Mo_2.77_,Al_0.23_)_12_ was established. The crystals were obtained by grinding in an agate mortar the reagents NaNO_3_, Cr(NO_3_)_3_·9H_2_O and (NH_4_)_6_Mo_7_O_24_·4H_2_O in a Na:Cr:Mo molar ratio of 1:1:4, respectively. The resulting mixture was calcined at 673 K to remove volatiles including NO_2_, NH_3_ and H_2_O. The residual powder thus obtained was finely ground and then returned to the oven at a temperature close to the melting point at 973 K for three days to promote germination and crystal growth. After cooling, crystals of parallelepipedal shape and optimal size for data collection were obtained. A crystal of a good quality, selected under a polarizing microscope, was used for intensity measurements

## Refinement   

**Figure 7:**
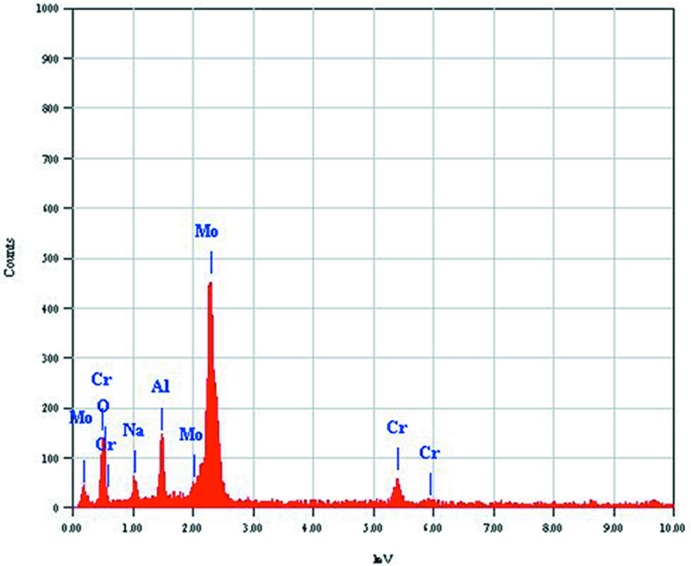
EDX analysis of the sample confirming the presence of aluminium in Na_0.72_(Cr_0.48_,Al_1.52_)(Mo_2.77_, Al_0.23_)O_12_.

Crystal data, data collection and structure refinement details are summarized in Table 3 ▸. After processing the data, the structure was solved successfully in the *R* 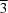 *c* space group, using direct methods implemented in the *SHELXS97* program (Sheldrick, 2008 ▸). The molybdenum, chromium and oxygen atoms were located first. At this stage, an empirical ψ-scan correction (North *et al.*, 1968 ▸) was applied. Difference-Fourier syntheses using the program *SHELXL97* (Sheldrick, 2008 ▸), allowed the rest of the atoms in the cell to be localized. We obtained intense peaks close to Cr and Mo; the liberation of the occupancy factors led to values different from the full site occupancy (0.62530 for Mo and 0.24035 for Cr). The EDX analysis (Fig. 7 ▸) of the sample confirmed the presence of aluminium and we then used EADP and EXYZ constraints as well as SUMP to refine Al1 with the Mo1 site and Al2 with the Cr1 site. After refinement and verification of the electrical neutrality, the final formula was Na_0.72 (1)_(Cr_0.48 (1)_,Al_1.52 (2)_)(Mo_2.77 (3)_,Al_0.23 (2)_)O_12_. The remaining maximum and minimum electron densities in the difference-Fourier map are acceptable and are at 0.78 Å from the Mo1 site and at 0.89 Å from the Mo2, respectively.

## Supplementary Material

Crystal structure: contains datablock(s) I. DOI: 10.1107/S2056989018003031/vn2134sup1.cif

Structure factors: contains datablock(s) I. DOI: 10.1107/S2056989018003031/vn2134Isup2.hkl

CCDC reference: 1824957

Additional supporting information:  crystallographic information; 3D view; checkCIF report

## Figures and Tables

**Table 1:** Selected geometric parameters (Å, °)

Mo1—O2^i^	1.7358 (15)	Cr1—O2^v^	1.9721 (16)
Mo1—O2	1.7359 (15)	Cr1—O2^vi^	1.9721 (16)
Mo1—O1	1.7540 (16)	Na1—O1^ii^	2.4987 (15)
Mo1—O1^i^	1.7540 (15)	Na1—O1^vii^	2.4987 (15)
Cr1—O1^ii^	1.9668 (16)	Na1—O1^iii^	2.4987 (15)
Cr1—O1^iii^	1.9668 (16)	Na1—O1^viii^	2.4987 (15)
Cr1—O1^iv^	1.9669 (16)	Na1—O1^iv^	2.4987 (15)
Cr1—O2	1.9720 (16)	Na1—O1^ix^	2.4987 (15)
			
O2^i^—Mo1—O2	109.56 (11)	O1^iv^—Cr1—O2^vi^	88.68 (7)
O2^i^—Mo1—O1	107.85 (8)	O2—Cr1—O2^vi^	91.35 (7)
O2—Mo1—O1	111.40 (8)	O2^v^—Cr1—O2^vi^	91.35 (7)
O2^i^—Mo1—O1^i^	111.40 (8)	O1^ii^—Na1—O1^vii^	180.0
O2—Mo1—O1^i^	107.86 (8)	O1^ii^—Na1—O1^iii^	65.74 (6)
O1—Mo1—O1^i^	108.80 (11)	O1^vii^—Na1—O1^iii^	114.26 (6)
O1^ii^—Cr1—O1^iii^	87.18 (7)	O1^ii^—Na1—O1^viii^	114.26 (6)
O1^ii^—Cr1—O1^iv^	87.18 (7)	O1^vii^—Na1—O1^viii^	65.74 (6)
O1^iii^—Cr1—O1^iv^	87.18 (7)	O1^iii^—Na1—O1^viii^	180.0
O1^ii^—Cr1—O2	92.79 (7)	O1^ii^—Na1—O1^iv^	65.74 (6)
O1^iii^—Cr1—O2	88.68 (7)	O1^vii^—Na1—O1^iv^	114.26 (6)
O1^iv^—Cr1—O2	175.86 (7)	O1^iii^—Na1—O1^iv^	65.74 (6)
O1^ii^—Cr1—O2^v^	88.68 (7)	O1^viii^—Na1—O1^iv^	114.26 (6)
O1^iii^—Cr1—O2^v^	175.86 (7)	O1^ii^—Na1—O1^ix^	114.26 (6)
O1^iv^—Cr1—O2^v^	92.79 (7)	O1^vii^—Na1—O1^ix^	65.74 (6)
O2—Cr1—O2^v^	91.35 (7)	O1^iii^—Na1—O1^ix^	114.26 (6)
O1^ii^—Cr1—O2^vi^	175.85 (7)	O1^viii^—Na1—O1^ix^	65.74 (6)
O1^iii^—Cr1—O2^vi^	92.79 (7)	O1^iv^—Na1—O1^ix^	180.0

Symmetry codes: (i) 
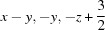
; (ii) 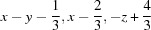; (iii) 
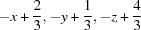
; (iv) 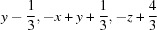; (v) 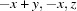; (vi) 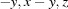; (vii) 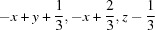; (viii) 
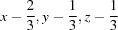
; (ix) 
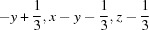
.

**Table 2:** CHARDI and BVS analyses for the cations in the Na_0.72_Cr_0.48_Al_1.76_Mo_2.77_O_12_ compound *q*(*i*) = formal oxidation number; sof(*i*) = site occupancy; CN(*i*) = classical coordination number; *Q*(*i*) = calculated charge; *V*(*i*) = calculated valence; ECoN(*i*) = coordination number; *d*_mean_(*i*) = mean distance; *d*_med_(*i*) = median distance.

Cation	*q*(*i*)·sof(*i*)	*Q*(*i*)	*V*(*i*)	CN(*i*)	ECoN(*i*)	*d*_mean_	*d*_med_
Mo1/Al1	5.78	5.77	5.8426	4	4.00	1.7448	1.7443
M(Cr1/Al2)	3.000	2.99	2.9397	6	6.00	1.9694	1.9696
Na1	0.72	0.71	0.6893	6	6.00	2.4989	2.4989

σ_cat_ is the dispersion factor for cationic charges where σ_cat_ = [Σ_*i*_(*q*_*i*_ − *Q*_*i*_)^2^/*N* − 1]^1/2^ = 0.025.

**Table 3:** Experimental details

Crystal data	
Chemical formula	Na_0.72_(Cr_0.48_·Al_1.52_)(Mo_2.77_·Al_0.23_)O_12_
*M*_r_	546.34
Crystal system, space group	Trigonal, *R* 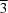 *c*
Temperature (K)	298
*a*, *c* (Å)	9.217 (2), 22.646 (2)
*V* (Å^3^)	1666.1 (7)
*Z*	6
Radiation type	Mo *K*α
μ (mm^−1^)	3.74
Crystal size (mm)	0.24 × 0.21 × 0.18

Data collection	
Diffractometer	Enraf–Nonius CAD-4
Absorption correction	ψ scan (North *et al.*, 1968 ▸)
*T*_min_, *T*_max_	0.491, 0.599
No. of measured, independent and observed [*I* > 2σ(*I*)] reflections	2878, 414, 401
*R*_int_	0.026
(sin θ/λ)_max_ (Å^−1^)	0.637

Refinement	
*R*[*F* ^2^ > 2σ(*F* ^2^)], *wR*(*F* ^2^), *S*	0.013, 0.024, 1.25
No. of reflections	414
No. of parameters	35
No. of restraints	2
Δρ_max_, Δρ_min_ (e Å^−3^)	0.23, −0.43

Computer programs: *CAD-4 EXPRESS* (Duisenberg, 1992 ▸; Macíček & Yordanov, 1992 ▸), *XCAD4* (Harms & Wocadlo, 1995 ▸), *SHELXS97* (Sheldrick, 2008 ▸), *SHELXL2014* (Sheldrick, 2015 ▸), *DIAMOND* (Brandenburg, 2006 ▸), *WinGX* (Farrugia, 2012 ▸) and *publCIF* (Westrip, 2010 ▸).

## supplementary crystallographic information

### Crystal data

**Table:**

Na_0.72_(Cr_0.48_·Al_1.52_)(Mo_2.77_·Al_0.23_)O_12_	*D*_x_ = 3.267 Mg m^−^^3^
*M**_r_* = 546.34	Mo *K*α radiation, λ = 0.71073 Å
Trigonal, *R*3*c*	Cell parameters from 25 reflections
*a* = 9.217 (2) Å	θ = 12.1–14.8°
*c* = 22.646 (2) Å	µ = 3.74 mm^−^^1^
*V* = 1666.1 (7) Å^3^	*T* = 298 K
*Z* = 6	Prism, red
*F*(000) = 1526	0.24 × 0.21 × 0.18 mm

### Data collection

**Table:**

Enraf–Nonius CAD-4 diffractometer	*R*_int_ = 0.026
Radiation source: fine-focus sealed tube	θ_max_ = 26.9°, θ_min_ = 3.1°
ω/2θ scans	*h* = −11→11
Absorption correction: ψ scan (North *et al.*, 1968)	*k* = −2→11
*T*_min_ = 0.491, *T*_max_ = 0.599	*l* = −28→28
2878 measured reflections	2 standard reflections every 120 reflections
414 independent reflections	intensity decay: 0.8%
401 reflections with *I* > 2σ(*I*)	

### Refinement

**Table:**

Refinement on *F*^2^	2 restraints
Least-squares matrix: full	*w* = 1/[σ^2^(*F*_o_^2^) + 4.1215*P*] where *P* = (*F*_o_^2^ + 2*F*_c_^2^)/3
*R*[*F*^2^ > 2σ(*F*^2^)] = 0.013	(Δ/σ)_max_ = 0.001
*wR*(*F*^2^) = 0.024	Δρ_max_ = 0.23 e Å^−^^3^
*S* = 1.25	Δρ_min_ = −0.42 e Å^−^^3^
414 reflections	Extinction correction: SHELXL2014 (Sheldrick, 2015), Fc^*^=kFc[1+0.001xFc^2^λ^3^/sin(2θ)]^-1/4^
35 parameters	Extinction coefficient: 0.00046 (6)

### Special details

**Table:**

Geometry. All esds (except the esd in the dihedral angle between two l.s. planes) are estimated using the full covariance matrix. The cell esds are taken into account individually in the estimation of esds in distances, angles and torsion angles; correlations between esds in cell parameters are only used when they are defined by crystal symmetry. An approximate (isotropic) treatment of cell esds is used for estimating esds involving l.s. planes.
Refinement. Refinement of F^2^ against ALL reflections. The weighted R-factor wR and goodness of fit S are based on F^2^, conventional R-factors R are based on F, with F set to zero for negative F^2^. The threshold expression of F^2^ > 2sigma(F^2^) is used only for calculating R-factors(gt) etc. and is not relevant to the choice of reflections for refinement. R-factors based on F^2^ are statistically about twice as large as those based on F, and R- factors based on ALL data will be even larger.

### Fractional atomic coordinates and isotropic or equivalent isotropic displacement parameters (Å^2^)

**Table:**

	*x*	*y*	*z*	*U*_iso_*/*U*_eq_	Occ. (<1)
Mo1	0.28488 (2)	0.0000	0.7500	0.01214 (9)	0.921 (6)
Al1	0.28488 (2)	0.0000	0.7500	0.01214 (9)	0.080 (10)
Cr1	0.0000	0.0000	0.63854 (3)	0.0109 (2)	0.238 (11)
Al2	0.0000	0.0000	0.63854 (3)	0.0109 (2)	0.761 (19)
Na1	0.0000	0.0000	0.5000	0.0259 (9)	0.724 (8)
O1	0.48491 (19)	0.17851 (19)	0.74736 (7)	0.0326 (4)	
O2	0.1686 (2)	−0.0152 (2)	0.68761 (6)	0.0376 (5)	

### Atomic displacement parameters (Å^2^)

**Table:**

	*U*^11^	*U*^22^	*U*^33^	*U*^12^	*U*^13^	*U*^23^
Mo1	0.01265 (11)	0.01229 (13)	0.01137 (12)	0.00614 (6)	0.00085 (4)	0.00169 (8)
Al1	0.01265 (11)	0.01229 (13)	0.01137 (12)	0.00614 (6)	0.00085 (4)	0.00169 (8)
Cr1	0.0117 (3)	0.0117 (3)	0.0092 (3)	0.00587 (14)	0.000	0.000
Al2	0.0117 (3)	0.0117 (3)	0.0092 (3)	0.00587 (14)	0.000	0.000
Na1	0.0322 (11)	0.0322 (11)	0.0132 (12)	0.0161 (6)	0.000	0.000
O1	0.0254 (8)	0.0218 (8)	0.0442 (9)	0.0071 (7)	−0.0038 (6)	0.0003 (7)
O2	0.0364 (9)	0.0446 (11)	0.0278 (8)	0.0172 (8)	−0.0093 (7)	0.0015 (7)

### Geometric parameters (Å, º)

**Table:**

Mo1—O2^i^	1.7358 (15)	Na1—O1^ii^	2.4987 (15)
Mo1—O2	1.7359 (15)	Na1—O1^vii^	2.4987 (15)
Mo1—O1	1.7540 (16)	Na1—O1^iii^	2.4987 (15)
Mo1—O1^i^	1.7540 (15)	Na1—O1^viii^	2.4987 (15)
Cr1—O1^ii^	1.9668 (16)	Na1—O1^iv^	2.4987 (15)
Cr1—O1^iii^	1.9668 (16)	Na1—O1^ix^	2.4987 (15)
Cr1—O1^iv^	1.9669 (16)	Na1—Al2^x^	3.1373 (7)
Cr1—O2	1.9720 (16)	Na1—Cr1^x^	3.1373 (7)
Cr1—O2^v^	1.9721 (16)	O1—Al2^iii^	1.9668 (16)
Cr1—O2^vi^	1.9721 (16)	O1—Cr1^iii^	1.9668 (16)
Cr1—Na1	3.1374 (7)	O1—Na1^xi^	2.4987 (15)
			
O2^i^—Mo1—O2	109.56 (11)	O1^iii^—Na1—O1^iv^	65.74 (6)
O2^i^—Mo1—O1	107.85 (8)	O1^viii^—Na1—O1^iv^	114.26 (6)
O2—Mo1—O1	111.40 (8)	O1^ii^—Na1—O1^ix^	114.26 (6)
O2^i^—Mo1—O1^i^	111.40 (8)	O1^vii^—Na1—O1^ix^	65.74 (6)
O2—Mo1—O1^i^	107.86 (8)	O1^iii^—Na1—O1^ix^	114.26 (6)
O1—Mo1—O1^i^	108.80 (11)	O1^viii^—Na1—O1^ix^	65.74 (6)
O1^ii^—Cr1—O1^iii^	87.18 (7)	O1^iv^—Na1—O1^ix^	180.0
O1^ii^—Cr1—O1^iv^	87.18 (7)	O1^ii^—Na1—Al2^x^	141.20 (4)
O1^iii^—Cr1—O1^iv^	87.18 (7)	O1^vii^—Na1—Al2^x^	38.80 (4)
O1^ii^—Cr1—O2	92.79 (7)	O1^iii^—Na1—Al2^x^	141.19 (4)
O1^iii^—Cr1—O2	88.68 (7)	O1^viii^—Na1—Al2^x^	38.81 (4)
O1^iv^—Cr1—O2	175.86 (7)	O1^iv^—Na1—Al2^x^	141.19 (4)
O1^ii^—Cr1—O2^v^	88.68 (7)	O1^ix^—Na1—Al2^x^	38.81 (4)
O1^iii^—Cr1—O2^v^	175.86 (7)	O1^ii^—Na1—Cr1^x^	141.20 (4)
O1^iv^—Cr1—O2^v^	92.79 (7)	O1^vii^—Na1—Cr1^x^	38.80 (4)
O2—Cr1—O2^v^	91.35 (7)	O1^iii^—Na1—Cr1^x^	141.19 (4)
O1^ii^—Cr1—O2^vi^	175.85 (7)	O1^viii^—Na1—Cr1^x^	38.81 (4)
O1^iii^—Cr1—O2^vi^	92.79 (7)	O1^iv^—Na1—Cr1^x^	141.19 (4)
O1^iv^—Cr1—O2^vi^	88.68 (7)	O1^ix^—Na1—Cr1^x^	38.81 (4)
O2—Cr1—O2^vi^	91.35 (7)	Al2^x^—Na1—Cr1^x^	0.0
O2^v^—Cr1—O2^vi^	91.35 (7)	O1^ii^—Na1—Cr1	38.80 (4)
O1^ii^—Cr1—Na1	52.76 (5)	O1^vii^—Na1—Cr1	141.20 (4)
O1^iii^—Cr1—Na1	52.76 (5)	O1^iii^—Na1—Cr1	38.81 (4)
O1^iv^—Cr1—Na1	52.76 (5)	O1^viii^—Na1—Cr1	141.19 (4)
O2—Cr1—Na1	124.30 (5)	O1^iv^—Na1—Cr1	38.81 (4)
O2^v^—Cr1—Na1	124.30 (5)	O1^ix^—Na1—Cr1	141.19 (4)
O2^vi^—Cr1—Na1	124.30 (5)	Al2^x^—Na1—Cr1	180.0
O1^ii^—Na1—O1^vii^	180.0	Cr1^x^—Na1—Cr1	180.0
O1^ii^—Na1—O1^iii^	65.74 (6)	Mo1—O1—Al2^iii^	144.66 (9)
O1^vii^—Na1—O1^iii^	114.26 (6)	Mo1—O1—Cr1^iii^	144.66 (9)
O1^ii^—Na1—O1^viii^	114.26 (6)	Al2^iii^—O1—Cr1^iii^	0.0
O1^vii^—Na1—O1^viii^	65.74 (6)	Mo1—O1—Na1^xi^	126.81 (8)
O1^iii^—Na1—O1^viii^	180.0	Al2^iii^—O1—Na1^xi^	88.43 (6)
O1^ii^—Na1—O1^iv^	65.74 (6)	Cr1^iii^—O1—Na1^xi^	88.43 (6)
O1^vii^—Na1—O1^iv^	114.26 (6)	Mo1—O2—Cr1	158.35 (10)

Symmetry codes: (i) *x*−*y*, −*y*, −*z*+3/2; (ii) *x*−*y*−1/3, *x*−2/3, −*z*+4/3; (iii) −*x*+2/3, −*y*+1/3, −*z*+4/3; (iv) *y*−1/3, −*x*+*y*+1/3, −*z*+4/3; (v) −*x*+*y*, −*x*, *z*; (vi) −*y*, *x*−*y*, *z*; (vii) −*x*+*y*+1/3, −*x*+2/3, *z*−1/3; (viii) *x*−2/3, *y*−1/3, *z*−1/3; (ix) −*y*+1/3, *x*−*y*−1/3, *z*−1/3; (x) −*x*, −*y*, −*z*+1; (xi) *x*+2/3, *y*+1/3, *z*+1/3.
